# Electrocardiographic changes in hiatal hernia: a case report

**DOI:** 10.4076/1757-1626-2-8278

**Published:** 2009-09-15

**Authors:** Gregoriana Zanini, Giuseppe Seresini, Marco Racheli, Monica Bortolotti, Adriana Virgillo, Adriana Novali, Claudia Benetello, Gian Franco Pasini

**Affiliations:** U.O. di Cardiologia, Ospedale di GavardoVia Gosa, Gavardo (Brescia)-Italy

## Abstract

We describe the case of a 78-year-old woman admitted to our department for suspected silent myocardial ischaemia with the evidence of T wave inversion in anterior lead. All the instrumental exams excluded inducible myocardial ischaemia. A gastroscopy showed a moderate hiatal hernia. We postulate that electrocardiogram modification could be attributed to hiatal hernia.

## Case presentation

**Figure 1.**
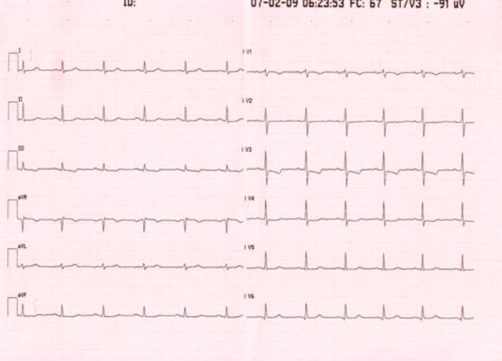
Electrocardiogram (ECG) showing T wave inversion in precordial leads V1-V3.

A 78-year-old Caucasian Italian woman was admitted to our department for suspected silent myocardial ischaemia in a recent electrocardiogram (ECG) showing T wave inversion in precordial leads V1-V3 (Figure 1). The patient had an history of hypertension in treatment with ace-inhibitors and hypercholesterolemia and she performed an ECG as a screening for hypertension; she had no history of myocardial infarction or angina pectoris, and there was no family history of ischaemic heart disease.

She had exertional dyspnoea for the past 1 year. On admission the patient was asymptomatic with a blood pressure of 130/85 mmHg, normal pulse heart rate in regular rhythm.

There was not significant alteration at the physical examination.

Haematological exam did not show significant alteration in particular no evidence of anaemia, kidney or hepatic disease. Electrolytes were normal and there wasn’t evidence of thyroid dysmetabolism.

The chest X-ray showed a normal heart shadow and the ECG demonstrated sinus rhythm with T wave inversion in leads V1-V3 (Figure 1).

**Figure 2.**
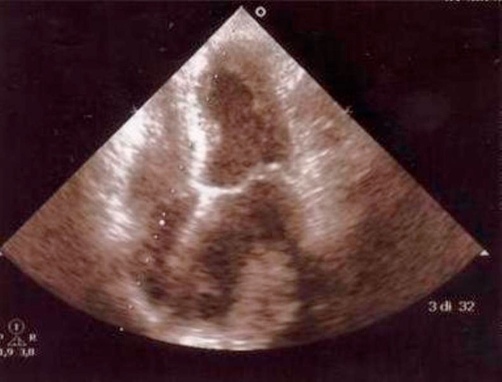
Transthoracic echocardiography (TTE) apical four chamber view shows an apparent left atrial “mass” in a dilated left atrium.

**Figure 3.**
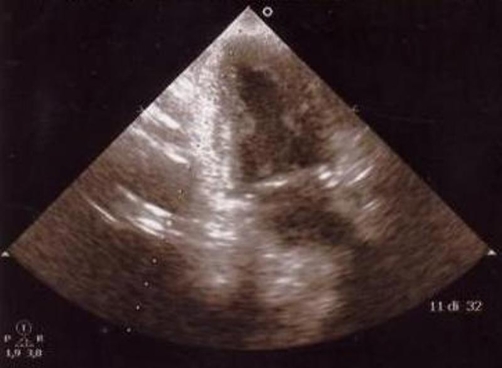
Transthoracic echocardiography (TTE) apical two chamber view.

Transthoracic echocardiography (TTE) revealed normal wall motion of all the left ventricle, no pulmonary hypertension and there wasn’t pericardial effusion. In addition way TTE revealed an apparent left atrial “mass” in a dilated left atrium, with its maximal size when the left atrium was imaged in a posterior plane, but smaller or absent in more anterior planes (Figures 2 and 3).

In the doubt of a giant hiatal hernia the patients was submitted to a gastroscopy that confirmed our suspicions.

Then the patient performed an exercise test that was maximal and negative for myocardial ischaemia. To confirm the diagnosis the patient was submitted to another inducible myocardial ischaemia test (Dipyridamole Myocardial Perfusion Scintigraphy) also negative.

We discharged the patient with hypotensive therapy (ACE-inhibitor) and gastro protection. We re-evaluated the patient after one month and she told us a sensible reduction in exert ional dyspnoea.

## Discussion

ECG specific abnormality in healthy women is frequent especially localized in lateral size but T wave inversion in precordial leads V1-V3 are very suspicious of ischaemia also in asymptomatic women.

We describe the case of a middle age women with poorly cardiovascular risk factors, asymptomatic for angina but symptomatic for dyspnoea. Her ECG abnormalities was suspicious of silent ischaemia but all tests made to detect inducible ischaemia were negative.

Transthoracic Echocardiography revealed a presence of a hiatal hernia like a left atrial mass that maybe the cause for these ECG abnormalities.

It was realized about 20 years ago that the sonographic appearance of a diaphragmatic hernia could simulate a left atrial mass [1]. Over the last 10 years, many reports of single cases of hiatal hernia have been appeared in the cardiac echocardiography literature [2], and only few instances of cardiac compression causing serious symptoms (like syncope or dyspnoea with recurrent heart failure or arrhythmia) have been reported [3-6].

Hokamaki et al. described the case of a women with the same age of our women admitted for chest pain and with dynamic ST-T wave changes due to a giant hiatal hernia. Surgical correction of the hiatal hernia restore ECG to normal [7].

Sonoda et al. Reported ST segment alteration during an oesophageal reconstruction surgery [8].

Siu et al. [5], demonstrated how a hiatal hernia could bring recurrent heart failure.

In conclusion there are very few cases in the literature about these ST segment alteration related to hiatal hernia but all are described in women and all in middle age women.

So we could postulate that in our case ST segment alteration and exertional dyspnoea may be due to the hiatal hernia.

The misdiagnosis of these pathology could make the physician to a round of complex and reiterated exams that due a sensible increase in healthy cost and sometimes to a uncorrected treatment.
